# Applications of Machine Learning (ML) in the context of marketing: a bibliometric approach

**DOI:** 10.12688/f1000research.160010.1

**Published:** 2025-01-15

**Authors:** Sebastián Cardona-Acevedo, Erica Agudelo-Ceballos, Diana Arango-Botero, Alejandro Valencia-Arias, Juana De La Cruz Ramírez Dávila, Jesus Alberto Jimenez Garcia, Carlos Flores Goycochea, Ezequiel Martínez Rojas

**Affiliations:** 1 Centro de Investigaciones, Institución Universitaria Escolme, Medellín, 50005, Colombia; 2 Departamento de Ciencias Administrativas, Instituto Tecnologico Metropolitano, Medellín, Antioquia, Colombia; 3 Escuela de Ingeniería, Universidad Senor de Sipan, Chiclayo, Lambayeque, Peru; 4 Departamento de Estudios Generales, Universidad Senor de Sipan, Chiclayo, Lambayeque, Peru; 5 Dirección de Planificación y Desarrollo Institucional, Universidad Senor de Sipan, Chiclayo, Lambayeque, Peru; 6 Instituto de Investigaciones y Estudios de la Mujer, Universidad Ricardo Palma, Santiago de Surco, Peru; 7 Vicerrectoría de Investigación e Innovación, Universidad Arturo Prat, Iquique, Tarapacá Region, Chile

**Keywords:** Personalization, Decision making, Artificial intelligence, Digital marketing, PRISMA-2020.

## Abstract

Currently, machine learning applications in marketing allow to optimize strategies, personalize experiences and improve decision making. However, there are still several research gaps, so the objective is to examine the research trends in the use of machine learning in marketing. A bibliometric analysis is proposed to assess the current scientific activity, following the parameters established by PRISMA-2020. Machine learning applications in marketing have experienced steady growth and increased attention in the academic community. Key references, such as Miklosik and Evans, and prominent journals, such as IEEE Access and Journal of Business Research, have been identified. A thematic evolution towards big data and digital marketing is observed, and thematic clusters such as “digital marketing”, “interpretation”, “prediction”, and “healthcare" stand out. These findings demonstrate the continued importance and research potential of this evolving field.

## Introduction

Over the years, there has been a growing interest in the application of machine learning to marketing. Machine learning, which refers to the use of algorithms that can learn from data and improve their performance over time without being explicitly programmed, has the potential to transform the way marketing is done by allowing marketers to analyze vast amounts of data and gain insights that were previously impossible. The combination of machine learning and marketing has led to the development of new tools and techniques for predicting consumer behavior, identifying customer segments, and optimizing marketing campaigns.

One of the areas where machine learning has shown great promise is in understanding consumer behavior on digital marketing platforms. In addition, machine learning can be used to analyze data from digital marketing platforms to understand consumer behavior and identify factors that contribute to consumer loyalty ( Sriram et al., 2022). This can help marketers develop more targeted marketing campaigns and improve customer retention.

The use of machine learning in the financial sector is growing. Machine learning has the potential to improve financial decision making by analyzing large amounts of data and identifying patterns that can be used to make more accurate predictions. Ahmed et al. (2022) conducted a bibliometric review of articles on artificial intelligence and machine learning in finance and found that there has been a significant increase in research in this area in recent years.

Machine learning can also be used to improve the performance of marketing campaigns. Langen and Huber (2023) used causal machine learning to evaluate and improve the performance of a coupon campaign. They found that the use of causal machine learning can lead to significant improvements in campaign performance by identifying causal relationships between marketing activities and outcomes. In addition, machine learning can be used to assess marketing behavior and identify factors that contribute to brand equity. Using machine learning to perform meta-customer mix brand equity analysis can help marketers identify factors that contribute to brand equity and optimize their marketing efforts accordingly ( Xu et al., 2022).

The use of machine learning in marketing is a topic of growing interest today. This is because the combination of machine learning and marketing has the potential to transform the way marketing strategies are implemented by enabling the analysis of large amounts of data and providing valuable information for decision making ( Ullal et al., 2021). Furthermore, Dai and Wang (2021) point out that machine learning can be used to predict customer response to marketing publications, allowing for better customer segmentation and personalization of marketing strategies. However, the application of machine learning in marketing also poses significant challenges, including the need to ensure the quality of data and the correctness of causal inferences ( Hair & Sarstedt, 2021). This includes the proper selection of variables and the understanding of the underlying causal effects; in this sense, the use of appropriate measurement and analysis techniques is essential.

On the other hand, the integration of machine learning and marketing also has ethical and privacy implications that must be carefully considered; it is important to ensure transparency and control over the data and information used in machine learning processes ( Ma & Sun, 2020). Likewise, it is necessary to consider the potential impact of these technologies on society and consumer decision-making.

The use of machine learning in the field of marketing has shown a significant increase, but there are still some important gaps in the research. Currently, more research is needed to explore the impact of big data and machine learning on the digital transformation of marketing ( Miklosik & Evans, 2020). In addition, it is necessary to identify more accurate machine learning techniques for the classification of targeted advertising ( Choi & Lim, 2020).

Finally, it is necessary to explore how machine learning can aid behavioral marketing research ( Hagen et al., 2020), although more research is needed that specifically examines this topic. In general, conducting a bibliometric on the use of machine learning in marketing would make it possible to identify the main research gaps and the areas that require more attention to help researchers better focus their efforts on developing new studies and analysis techniques. Therefore, the objective is to examine the research trends in the use of machine learning in marketing, allowing the construction of a research agenda focused on the materialization of future research, for which the following research questions are asked:

- What are the years when there has been more interest in the use of machine learning in marketing?
- How is the number of scientific articles on the use of machine learning in marketing growing?
- What are the main research references on the use of machine learning in marketing?
- What is the thematic evolution derived from the scientific production on the use of machine learning in marketing?
- What are the main thematic clusters on the use of machine learning in marketing?
- What are the growing and emerging keywords in the research field of the use of machine learning in marketing?
- Which topics are positioned as protagonists for the design of a research agenda on the use of machine learning in marketing?

This research has an introductory section that allows the analysis of the definition, importance and problems related to the use of machine learning in marketing. Next, there is the methodology that defines the parameters with which the bibliometric analysis of this research will be developed, then there are the results associated with the present study, the discussion that raises the existing gaps in the research and the design of the research agenda; finally, the conclusions related to the main aspects of this study are found.

## Methods

Based on the proposed research objective, it is proposed to carry out a bibliometric analysis as an exploratory methodology that will facilitate the evaluation of the scientific activity currently available ( Vera et al., 2022). It is important to emphasize that, in order to create clear and replicable methodologies, this research complies with the parameters established by the international PRISMA declaration in its version 2020, which defines the eligibility criteria, information sources, search strategy and data management, which are extremely important resources for the correct development of the methodological design, as has been demonstrated ( Page et al., 2021).

### Eligibility criteria

According to the PRISMA 2020 statement, the eligibility criteria are divided into: inclusion and exclusion criteria. For the inclusion criteria, it is important that both the title and the keywords contain the terms marketing and machine learning (automatic learning) or their synonyms, as this makes it easier to obtain documents that contain relevant information for this study.

The exclusion criteria are divided into three consecutive phases of document elimination, following the recommendations of the PRISMA 2020 statement. First, after reading the titles of the documents, all those documents with incorrect indexing are discarded, then those documents that prevent access to the full texts are excluded, although this does not apply to the bibliometric analysis, since it only analyzes metadata such as titles and keywords. Finally, documents that are the result of conference proceedings are eliminated, as well as documents that contain irrelevant information that delays the analyses that allow the objective of the study to be achieved.

### Sources of information

Web of Science and Scopus were selected as secondary information sources for the search of relevant documents for this study; both databases are frequently used by researchers from different countries, which makes them leading databases for information search ( Zhu & Liu, 2020).

### Search strategy

In order to guarantee an effective document search, this study follows the recommendations of PRISMA 2020, which specifies the importance of developing an adequate search method depending on the information source to be used; based on the above, two similar search equations were designed, slightly different to adapt to the needs of each of the databases. In this sense, the following search equations are available:

For the Scopus database:(TITLE((marketing OR advertising)AND“machinelearning”))

For theWebof Science database:(TI=((“marketing”OR“advertising”)AND“machinelearning”)))

### Data management

After the implementation of the search strategy, a total of 180 documents related to the use of machine learning in marketing were obtained, ranging from the year 2006 to the present year 2023, of which 122 belong to Scopus and 58 to Web of Science; these documents were stored and processed with the help of the tool Microsoft Excel ^®^, where the three phases of elimination previously defined were applied, where 122 articles were obtained to be analyzed in detail, in addition, with the help of the free software VOSviewer ( Van Eck and Waltman, 2011), the indicator graphs were created. bibliometric, which will facilitate the data analysis.

### Selection process

According to the PRISMA 2020 statement, as mentioned in Page et al. (2021), it is essential to include information on the use of an internal automatic classifier to assist in the selection process, as well as internal or external validation to assess the risk of lost studies or incorrect classifications. In this study, Microsoft Excel ^®^ automation tools were used as an internal resource; these tools were developed by all participating researchers, who in turn used them independently during the application of the inclusion and exclusion criteria; in this way, the risk of missing studies or incorrect classifications was minimized through the convergence of the results obtained.

### Data collection process

The present bibliometrics was carried out to analyze the applications of machine learning in the field of marketing. Following the guidelines proposed by Page et al. (2021), the methods used to collect the data from the selected reports were specified, in this sense, Microsoft Excel ^®^ was used as an automated tool for the data collection process from the two selected databases, all the authors of the study performed the role of independent reviewers, participating in the validation of the data, to guarantee the precision and reliability of the results, a process of confirmation of the data was carried out collectively, in which an absolute convergence of the results was sought. results obtained.

### Data elements

Two main approaches were used in this study: Firstly, an exhaustive search was carried out in different databases, using specific search equations designed for each one; this implied the inclusion of all articles that specifically mentioned Machine Learning applications in marketing, covering different measures, time points and analyses related to the topic; secondly, the relevance of the results obtained was taken into account, excluding those texts that presented missing or unclear information, since these do not contribute to an adequate understanding of the knowledge base on the subject of the study; in this way, the aim was to ensure consistency and coherence with the objectives and scope of the investigation.

### Assessment of the risk of bias of the study

This review required a rigorous assessment of the risk of bias in the included studies, which included specification of the methods used, including details of the tools used, the number of reviewers involved and whether they worked independently, as well as information on the automation tools used in the process.

In this particular study, the data collection process was carried out by all the authors of the research, following the same methodology, and the risk of bias assessment was also carried out using an automated tool in Microsoft Excel ^®^, this approach was chosen to ensure the quality and integrity of the results obtained, the automated Microsoft Excel ^®^ tool was used consistently by all the authors, which ensured a consistent and objective assessment of the studies included in the bibliometrics.

This methodological approach was based on the use of standardized and widely accepted risk of bias assessment tools in the scientific community. In addition, the use of an automated tool allows a more efficient and consistent assessment of a large number of studies, which contributes to the objectivity and robustness of the bibliometric results.

### Effect measures

The present investigation focused on carrying out a bibliometric on the applications of machine learning in the field of marketing, as part of this analysis, the measure of effect used in the synthesis or presentation of the results is specified for each result, it is important It should be noted that effect measures, such as the risk ratio or the difference in means, are more common in primary research, however, in this study based on secondary research sources, alternative measures were used, the number of publications and the number of citations related to the subject were analyzed. To carry out these analyses, Microsoft Excel ^®^ was used, as well as VOSviewer ^®^ to determine the thematic association between the existing nodes in the field of study.

### Synthesis methods

Within the framework of this research on the applications of machine learning in the field of marketing, different processes were carried out for the selection of eligible studies, the preparation of the data and the presentation of the results. To determine the studies included in the synthesis, predefined criteria were applied, such as the comparison of the characteristics of the intervention of each study with the groups planned for each synthesis, In the same way, methods were used for the preparation of the data, such as the management of statistics, missing abstracts and data conversions, thus ensuring the consistency and reliability of the results obtained, methods were also used to tabulate and visualize the results of individual studies and syntheses, allowing a clear and concise understanding of the results. In this research, bibliometric indicators of quantity, quality and structure were carried out, following the approach described by Durieux and Gevenois (2010), it should be noted that these indicators were automatically applied using Microsoft Excel ^®^ to all those documents that passed the three phases of exclusion.

### Assessment of reporting bias

In the bibliometric context of the applications of machine learning in marketing, the assessment of the risk of bias due to the lack of results in a synthesis (derived from reporting bias) implies considering any method used for this purpose, for which it is important to note that there may be a bias towards certain synonyms found in thesauri such as the IEEE, which may influence the inclusion criteria, the search strategy and the data collection, as well as the exclusion of irrelevant documents as exclusion criteria. There is a risk of omitting valuable information that could contribute to knowledge building on the topic in question.

### Assessment of certainty

In this study, the certainty of the body of evidence has been evaluated in a general way, in contrast to the primary studies that evaluate the certainty individually, the evaluation has been carried out through the independent application of inclusion and exclusion criteria, as well as in the definition of bibliometric indicators, likewise, the reporting of possible biases defined in the methodological design has been taken into account, and the limitations of the study are mentioned in the discussion phase, these approaches allow a more complete evaluation of the certainty in the body of evidence, providing a comprehensive perspective of the results obtained in the applications of Machine Learning in Marketing.

## Results

In this phase of the study, the results are presented according to the objective and the research questions, the bibliometric aspects related to Machine Learning in Marketing are detailed, and the elements related to research trends are examined, considering the type of growth. which presents the number of scientific articles related to the subject of study, the years of most interest on the part of the scientific community and the main research references.

**Figure 1.**
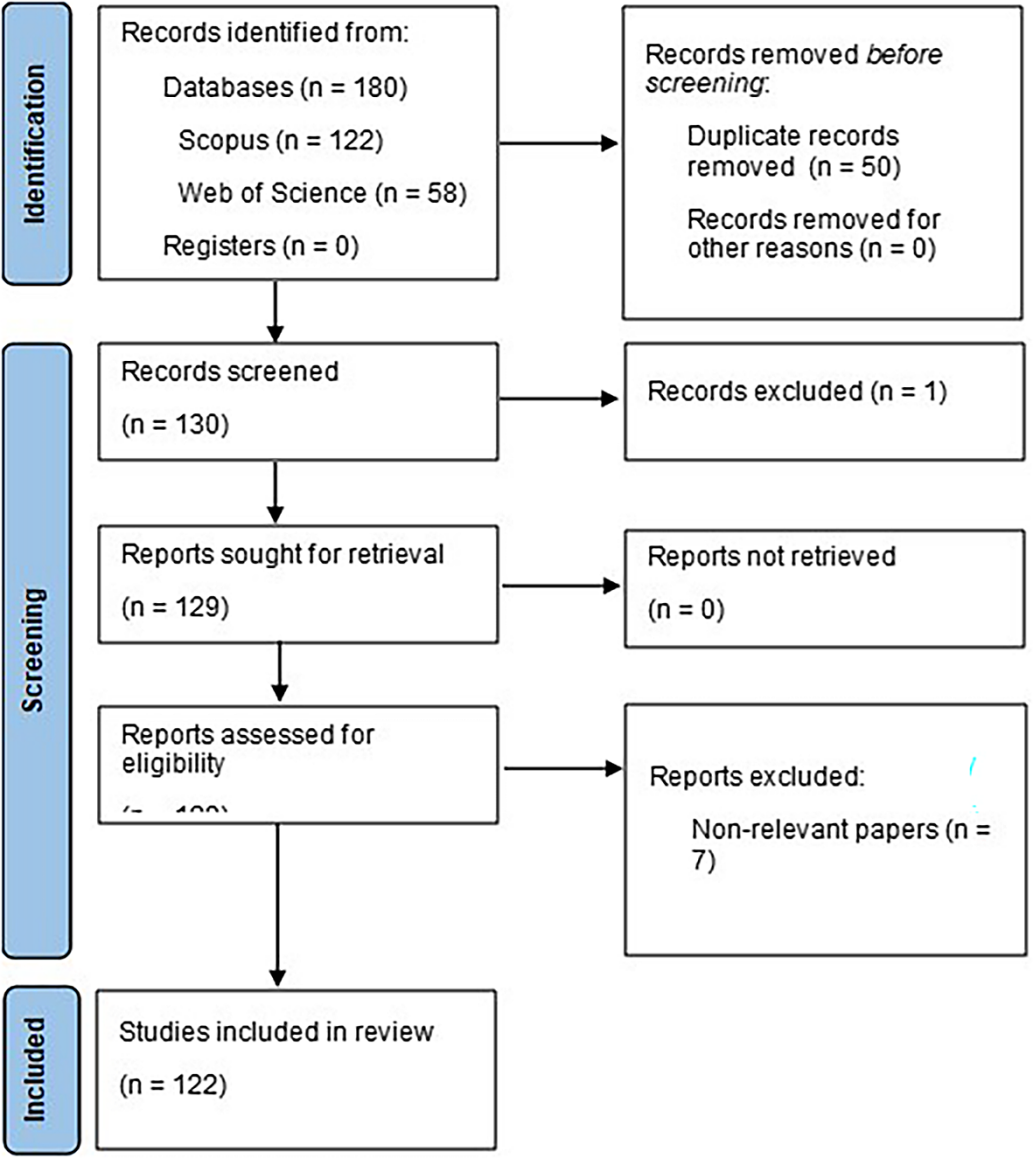
PRISMA flow chart.

In accordance with the above, Figure 1 shows a notorious exponential growth of 95. 41% in the publication of scientific articles on the applications of Machine Learning in marketing, it is observed that the most outstanding years in terms of the production of investigations on this subject were 2019, 2020, 2021 and 2022, the results reflect a sustained and accelerated increase in interest and research in this field in recent years, proving the growing recognition of the importance and potential of Machine Learning in the field of marketing, these results are particularly relevant considering an observation window that covers from 2006 to 2023, since they indicate a marked upward trend in the generation of scientific knowledge in this field during this period.

**Figure 2.**
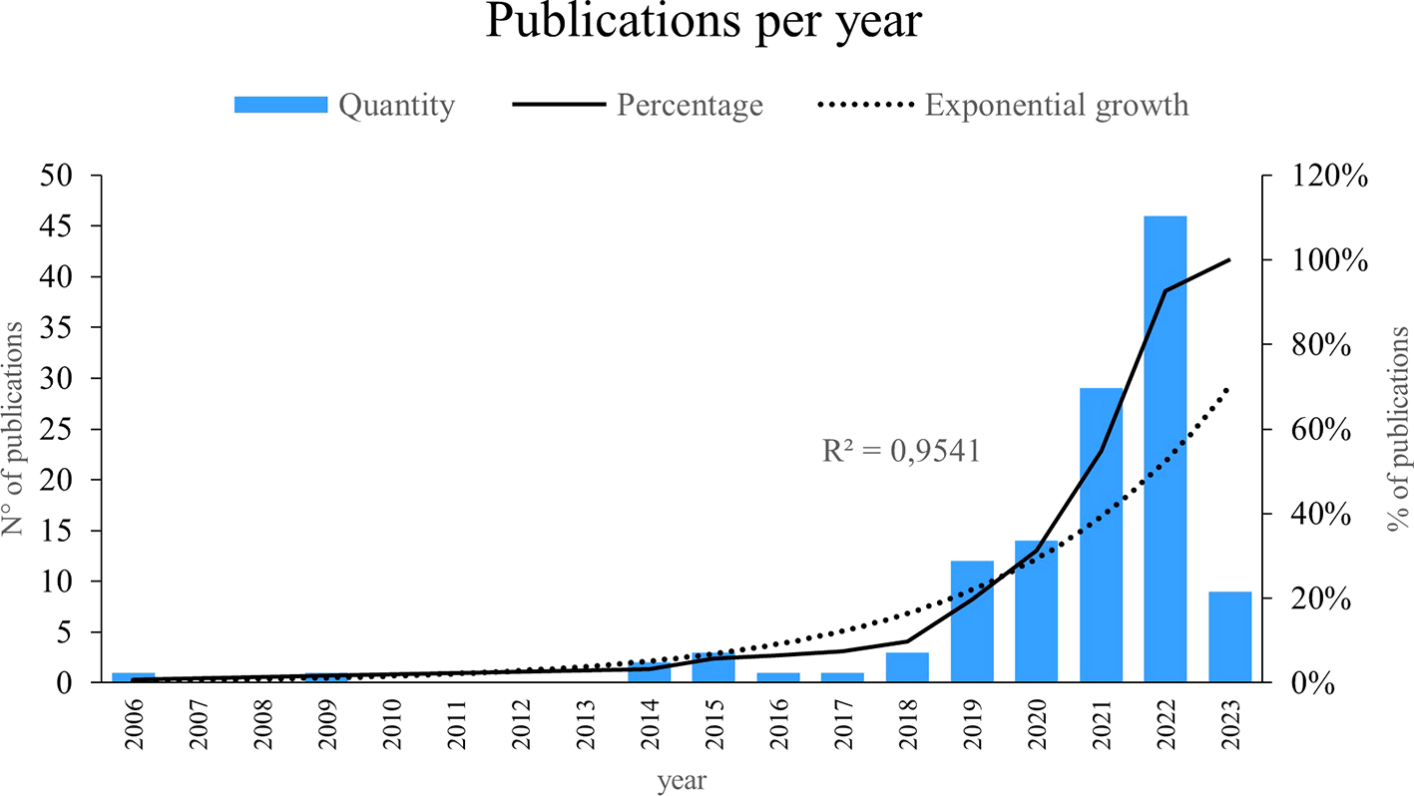
Publications per year.

With respect to studies on machine learning applications in marketing, Figure 2 shows three groups of prominent authors: First, there are Miklosik and Evans, who stand out for both their high scientific productivity and their impact on the academic community; second, there is the group of authors led by Sun, Ma, Wong, Lui, and Cui, who stand out mainly for the impact of their research, despite having a low index of scientific productivity; finally, the group of authors led by Zang and Wang is identified, who stand out for their high scientific productivity, although their number of citations is not so significant. These results highlight the diversity of approaches and results in the field of machine learning applications in marketing, where different authors stand out for their productivity, impact, or a combination of both.

**Figure 3.**
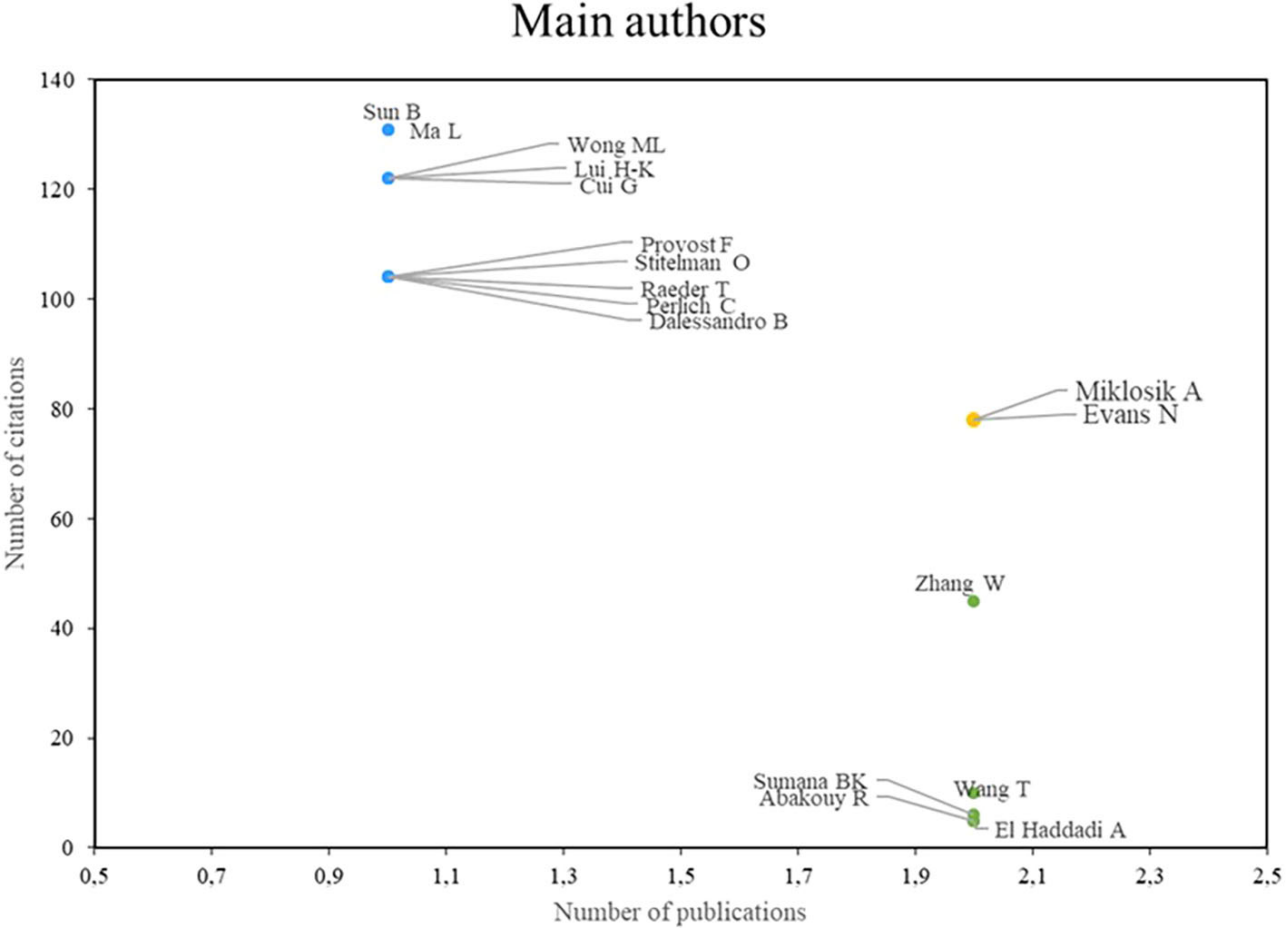
Main authors.

With regard to the most important journals, Figure 3 is sectorized by points; the yellow ones are the most referenced, since they are among those that publish the most and have been cited the most; the blue ones, which are impact journals, do not publish much but are the most cited; and the green ones correspond to the most productive journals, although they have not been cited much, are those that are most interested in the subject or tend to publish the most on it. Consequently, three groups of outstanding scientific journals have been identified: The first group is characterized by high productivity and impact, and includes journals such as IEEE Access and Journal of Business Research; the second group, although it has a low index of scientific productivity, stands out for its impact, and includes journals such as the International Journal of Research in Marketing and Management Science; finally, the third group, which is characterized by its scientific productivity and not so much by the number of citations, is mainly led by the ACM International Conference Proceeding Series. These results highlight the diversity of approaches and the strengths of the most relevant scientific journals in the field of machine learning applications in marketing.

**Figure 4.**
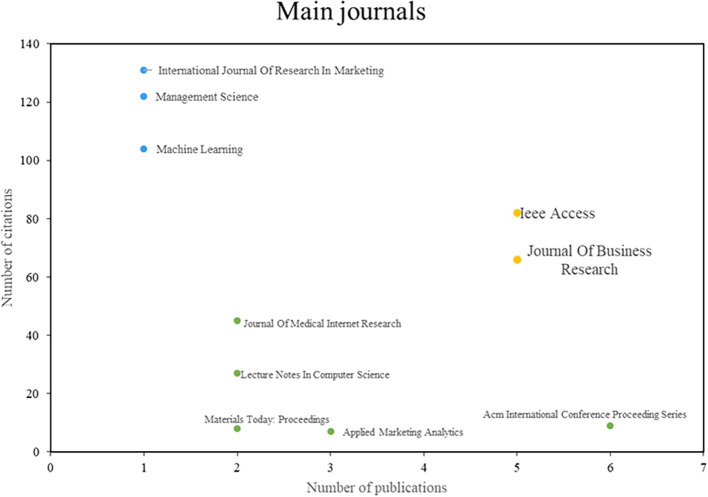
Main journals.

In the field of machine learning applications in marketing, Figure 4 shows the most outstanding countries in terms of the topic, listing the most impactful, productive and relevant countries. They stand out in terms of both the number of publications and the number of citations. In this sense, three groups of outstanding countries are identified: Firstly, there are those that stand out both in terms of scientific productivity and impact, with the United States being the main reference in this group; secondly, there are countries that stand out in terms of impact despite having a low index of scientific productivity, with the participation of authors from Hong Kong standing out; finally, a group of countries is identified that stand out mainly for their scientific productivity and not so much for the number of citations received, with India and China being highlighted in particular. These results show the diversity of approaches and strengths in research on the applications of machine learning in marketing at a global level.

**Figure 5.**
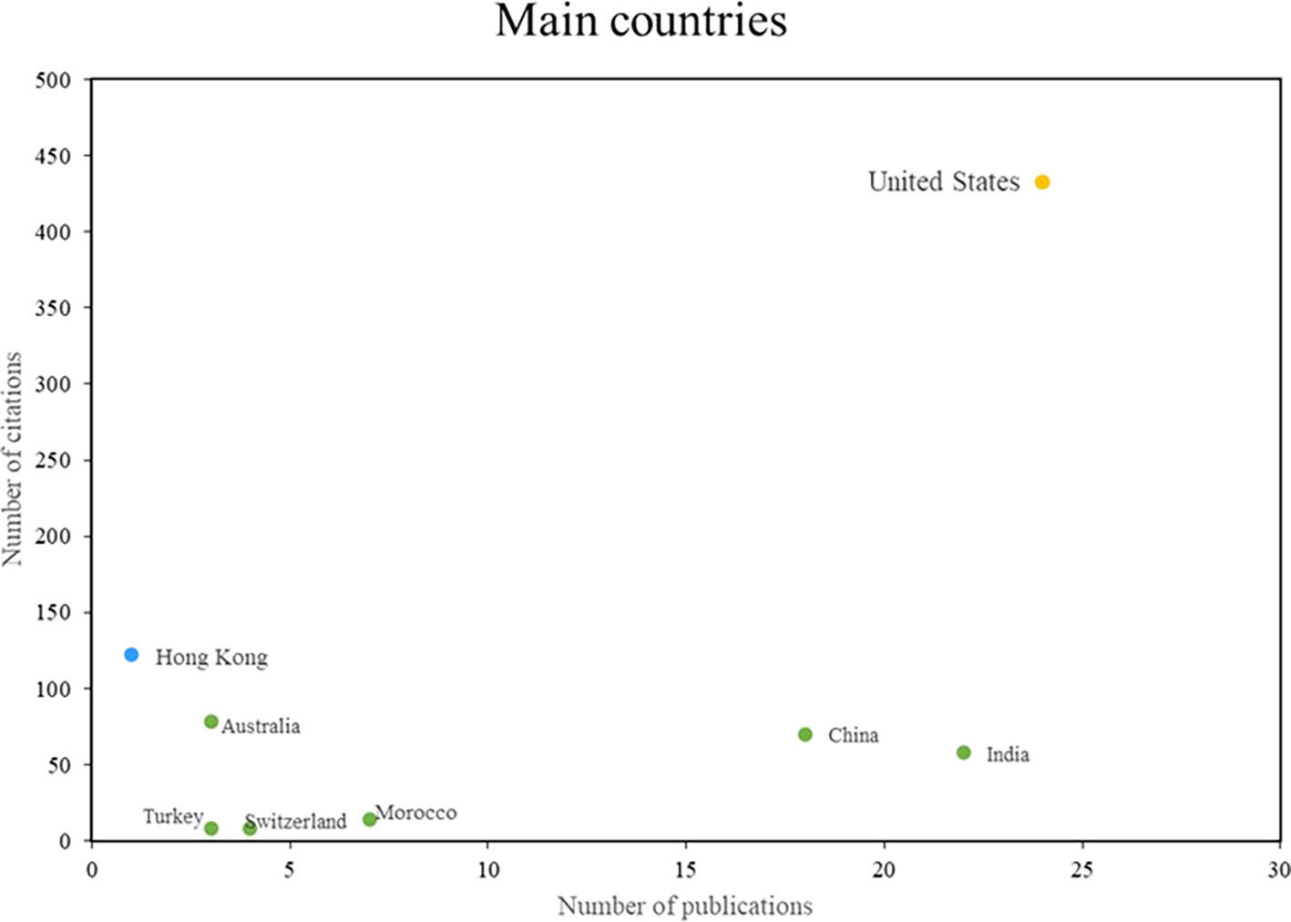
Main countries.

Once the results in terms of scientific production have been analyzed, the thematic elements are considered, tracking the activity related to the main ideas, such as research trends and the most relevant words. Figure 5 shows an analysis of the evolution of the literature between 2006 and 2023, it was observed that in 2006, as the first year of analysis, relevant concepts such as Evolutionary Programming emerged, likewise in recent years shows a notable predominance of topics such as Neural Networks, Big Data and Digital Marketing, reflecting current research trends in this area.

**Figure 6.**
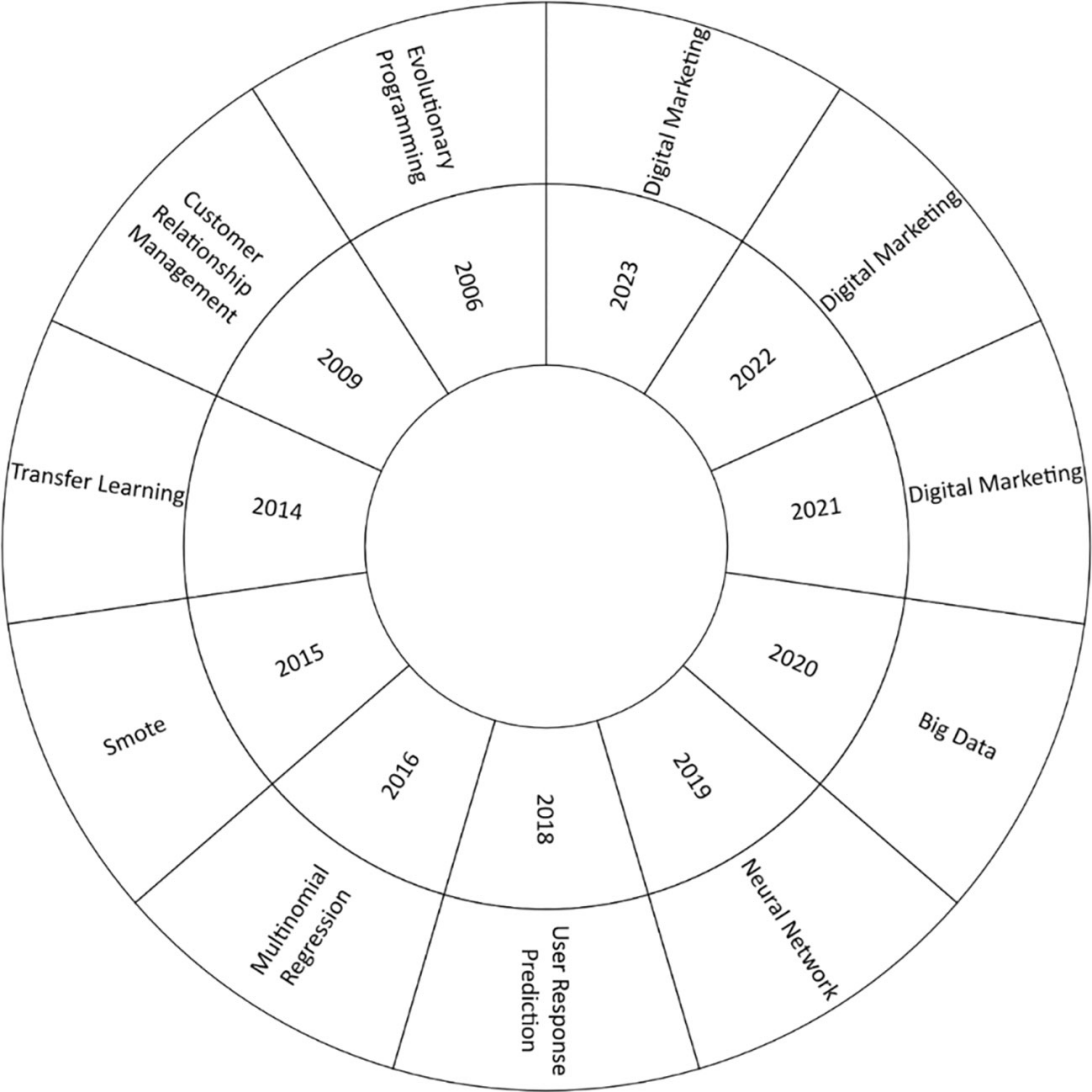
Thematic evolution.

Figure 6 describes the association between concepts based on thematic clusters of different colors, highlighting the most relevant ones and the words related to each one, giving rise to new investigations based on them. The main keyword co-occurrence network is presented organized in 6 thematic clusters, the light blue cluster stands out as the most prominent, composed of terms such as “digital marketing”, “interpretation”, “prediction” and “healthcare”, followed by the dark blue cluster, which includes terms such as “big data”, “artificial intelligence”, “marketing strategy”, “social media” and “customer churn”, in addition to other clusters of red, purple, yellow and green, which reveal additional elements of conceptual affinity in the field studied.

**Figure 7.**
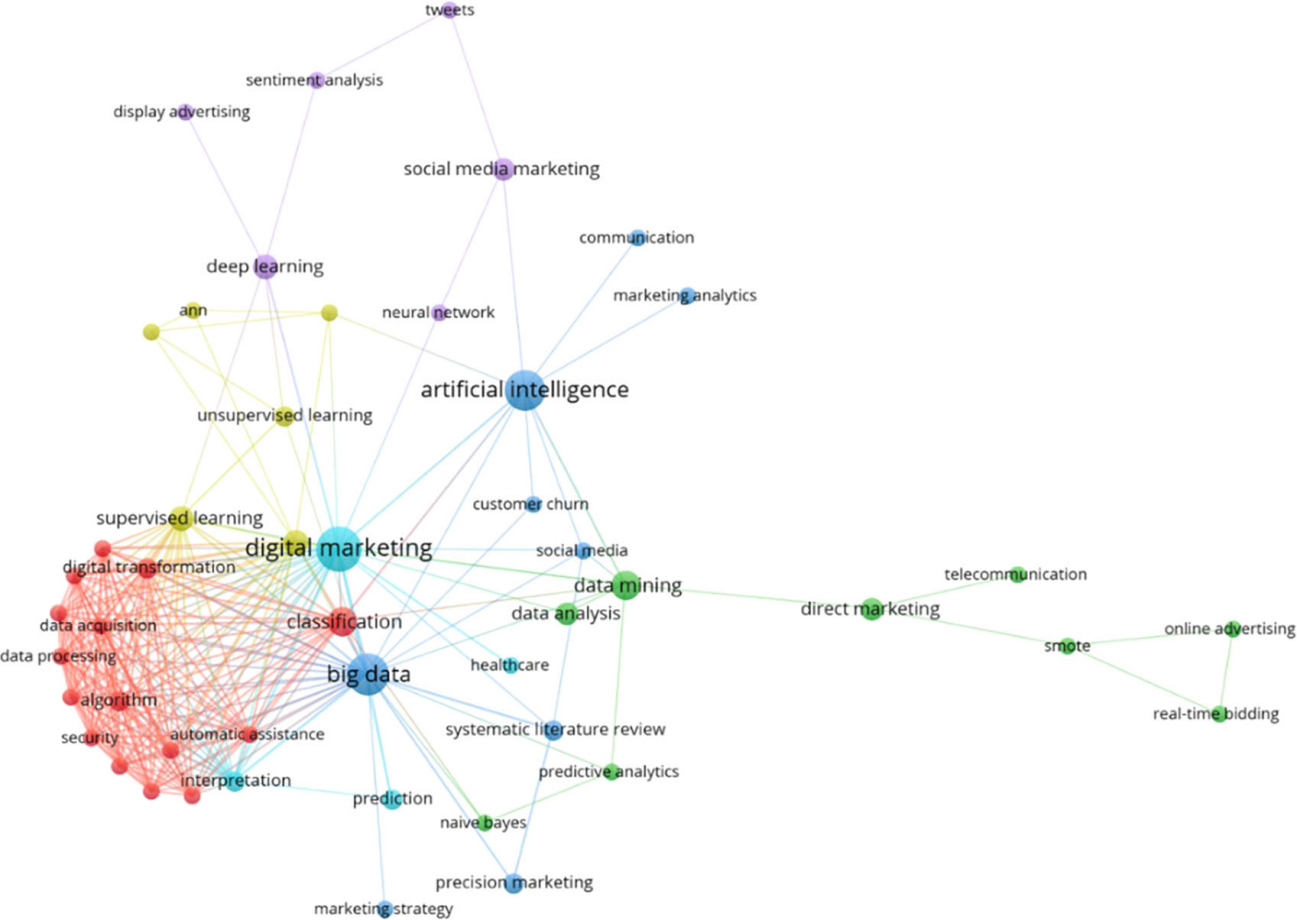
Keyword co-ocurrence network.

The present investigation proposes in Figure 7, an approach based on a Cartesian plane that allows analyzing the relationship between the frequency of use of keywords on the X-axis and the validity of their use on the Y-axis, which provides a clear vision. and structured analysis of bibliometric data in the field of machine learning applications in marketing.

This approach allows us to identify four different quadrants in the plan: In quadrant 2, there are keywords that are rare but highly topical, which makes them emergent and relevant in the current context; some examples of these keywords are “Healthcare”, “Systematic Literature Review”, “Algorithm”, “Automatic Assistance” and “Consumer Segmentation”; on the other hand, consolidated and growing concepts, such as “Digital Marketing”, “Artificial Intelligence” and “Big Data”, are located in quadrant 1; It is important to point out that there is a fourth quadrant where the declining concepts are located, that is, those keywords that do not show evidence of use in the scientific literature studied, and a third quadrant that houses the concepts that are not fully consolidated in the field.

## Discussion

The discussion section of the results is presented in which it is proposed, in principle, an analysis of the main results in terms of the annual scientific production, the main research references, the thematic evolution, the thematic clusters and the observation of frequency and validity. of keywords, Next, the classification of the most significant keywords according to their function is proposed, the practical implications, limitations and identified research gaps are considered; Finally, the future research agenda on the use of Machine Learning in marketing is postulated.

### Analysis of the growth of scientific literature on the use of machine learning in marketing

As can be seen in Figure 2, research on the use of Machine Learning in marketing has been conducted between 2006 and 2023, revealing its importance in the number of investigations in each mentioned period. The year 2020 witnessed a significant increase in research on the use of machine learning in the field of marketing. In the same year, an investigation titled “Machine Learning in Marketing: Overview, Learning Strategies, Applications and Future Developments”, which provided a comprehensive overview of the topic, examining various learning strategies and relevant practical applications ( Brei, 2020); highlighting the ability of machine learning to optimize and personalize marketing strategies, covering areas such as market segmentation, consumer behavior prediction, product recommendation, sentiment analysis, and customer experience management. In addition, new machine learning approaches and techniques, such as deep learning and reinforcement learning, have been explored to further improve the accuracy and effectiveness of marketing predictions and recommendations.

In 2021, outstanding research was conducted on the use of machine learning in marketing. In one of these investigations, a machine learning-based framework was developed using post-marketing surveillance data to predict the outcomes of a therapy in patients with gynecological cancer, so that by targeting target patients, it could increase its efficacy and safety ( Liu et al., 2021), highlighting the application of machine learning in predicting therapeutic outcomes in the field of medical care. On the other hand, the study “Complexity construction of intelligent marketing strategy based on mobile computing and machine learning simulation environment” has an approach based on mobile computing and machine learning simulation to build intelligent marketing strategies in a complex environment ( Mao & Huang, 2021). This research highlights the importance of machine learning in building adaptive and sophisticated marketing strategies.

The research conducted in 2022 was important, including a comparative study titled “The derived demand for advertising expenses and implications on sustainability: A comparative study using deep learning and traditional machine learning methods”, in which traditional machine learning and deep learning methods were used to analyze the derived demand for advertising expenses and its implications on sustainability ( Birim et al., 2022). This study highlights the application of machine learning in understanding the dynamics of demand and its relationship with sustainability in the context of advertising. In addition, another study conducted an analysis to understand the role of Machine Learning, robotics, and artificial intelligence in digital marketing, highlighting the importance of these emerging technologies in optimizing digital marketing strategies ( Boddu et al., 2022).

In 2023, a study was conducted on consumer behavior in digital marketing platforms, especially in terms of consumer loyalty using machine learning, which highlights the application of machine learning in the study of consumer behavior and its relationship with loyalty in the context of digital marketing platforms, also highlighting the importance of using machine learning techniques to better understand consumer preferences and behavior in digital environments, which can help improve marketing strategies. marketing and customer loyalty ( Sriram et al., 2022). This is one of the most outstanding investigations on the use of machine learning in the field of marketing for this year.

### Analysis of research references on the use of machine learning in marketing

As shown in Figure 3, the authors Miklosik A and Evans N stand out as recognized benchmarks in terms of both scientific productivity and academic impact, as both have a high number of publications and have made significant contributions to the field. In a joint publication, they analyzed the impact of big data and machine learning on digital transformation in marketing ( Miklosik & Evans, 2020), providing a comprehensive view of the implications and applications of these technologies, offering valuable insights for researchers and marketing professionals.

Among the authors who, although they are not considered benchmarks in terms of scientific productivity because they have fewer publications, the authors Sun B, Ma L, Wong ML, Lui H-K and Cui G, are authors who have left a significant mark in the field of the use of machine learning in marketing thanks to the academic impact they have generated through their research, among the contributions is a research that addresses the connection between computational power and human knowledge in the field of marketing, providing a valuable perspective on how machine learning and artificial intelligence can improve marketing strategies ( Ma & Sun, 2020). On the other hand, the publication “Machine learning for direct marketing response models: Bayesian networks with evolutionary programming” was based on the development of direct marketing response using machine learning, emphasizing the importance of these techniques in analyzing consumer response ( Cui et al., 2006).

Although Wang is not considered a benchmark in terms of scientific impact due to his lower number of citations, he has managed to stand out as a benchmark in terms of scientific productivity due to his large number of publications, his contributions in the field of the use of Machine Learning in marketing have been valuable, they have addressed relevant issues, and in addition, they provide new perspectives for the academic community and professionals in the sector. In the study “Evaluating the effectiveness of marketing campaigns for malls using a novel interpretable machine learning model”, he studies the evaluation of the effectiveness of marketing campaigns in shopping malls using an interpretable machine learning model ( Wang et al., 2022), providing an important analytical tool for marketers. In addition, his research on predicting customer engagement behavior in response to marketing posts using machine learning techniques ( Dai & Wang, 2021) provides valuable insights for understanding and improving the interaction between brands and consumers in the digital environment.

Similarly, in this study, an analysis of the main scientific journals in the field of study was carried out with the aim of identifying the most outstanding research references. In addition, aspects related to productivity and scientific impact are evaluated, as shown in Figure 4.

The journals IEEE Access and Journal of Business Research are recognized as outstanding leaders in terms of scientific production and academic impact in the field of machine learning applications in marketing. IEEE Access has published a study that focuses on segmentation and marketing strategies driven by machine learning in the context of online charitable giving in Taiwan ( Hsu et al., 2021). They also conduct a review of existing literature in the area of machine learning and marketing. These studies demonstrate the impact and importance of IEEE Access in the generation of knowledge in the field, underscoring its relevance ( Duarte et al., 2022).

In addition, the Journal of Business Research has played a pivotal role in providing valuable contributions to the field of marketing and machine learning; its publications examine the drivers, barriers, and future advancements of artificial intelligence and machine learning in management. of marketing, providing a comprehensive vision of this topic ( Volkmar et al., 2022). It also describes the creation of a real-time repository in the KNIME Hub for marketing applications using machine learning techniques. These articles show how the Journal of Business Research has had a significant impact and a notable influence on the dissemination of knowledge and the implementation of machine learning techniques in the field of marketing ( Ordenes & Silipo, 2021).

Although the International Journal of Research in Marketing and Management Science do not stand out for their scientific productivity in terms of the number of publications, they are widely recognized as leading academic journals due to the significant impact of their articles, as evidenced by the high number of citations received. The Orders study in the International Journal of Research in Marketing presents the development of a live repository for machine learning-based marketing applications at the KNIME Hub. This study demonstrates the importance and academic impact of the journal in the dissemination of knowledge and implementation of machine learning techniques in the field of marketing ( Ordenes & Silipo, 2021).

On the other hand, Management Science presents a study on the use of Bayesian networks combined with evolutionary programming based on machine learning to develop direct marketing response models ( Cui, Wong & Lui, 2006). This study highlights the relevance and impact of management science in the production of knowledge in the field of machine learning applied to marketing, as well as its academic influence in the scientific community.

Although the journal ACM International Conference Proceeding Series is not characterized by a high scientific impact in terms of citations, its scientific productivity is recognized due to the considerable number of publications that address the field of machine learning applied to marketing. The paper presented at the 4th International Conference on Smart City Applications examines the relevance of data-driven marketing and how machine learning can enhance the decision-making of marketers ( Abakouy et al., 2019).

In addition, the paper presented in the Proceedings of the 3rd International Conference on Information Management and Management Science, examines the relationship between vehicle registration data and vehicle marketing parameters by applying clustering techniques and machine learning based on IN rules ( Dahl et al., 2020). This study shows the importance and impact of the ACM International Conference Proceeding Series journals in generating knowledge in the field of marketing, especially regarding the application of machine learning.

On the other hand, this bibliometric analysis examines the main international references in the scientific production of the use of machine learning in marketing (see Figure 5). Among these references, the United States stands out in terms of both scientific productivity and academic impact, due to the high number of publications and citations generated in this field. An important study in this context presents a conceptual framework and sets a research agenda to understand the application of machine learning in the field of marketing ( Ngai & Wu, 2022), this contribution represents a milestone in current knowledge and sets future directions. of research in this area.

In addition, another article addresses the issue of algorithmic bias in marketing models based on Machine Learning, highlighting the importance of considering and addressing the biases inherent in these models ( Akter et al., 2022). This research strengthens the position of the United States as a generator of critical knowledge for the ethical and effective development of Machine Learning applications in the field of marketing.

Although Hong Kong is not recognized as a benchmark in terms of scholarly productivity in the use of machine learning in marketing, it stands out as one of the main benchmarks in terms of scholarly impact due to the high number of citations. A prominent example of Hong Kong’s contribution in this area is a study exploring the use of Bayesian networks with evolutionary programming to develop direct marketing response models using machine learning techniques ( Cui, Wong & Lui, 2006); this research has been widely cited and has generated significant impact in the field, which positions Hong Kong as a benchmark in the application of machine learning in marketing strategies; Although the number of publications in Hong Kong may be lower compared to other places, the quality and relevance of the research conducted demonstrates its academic impact and contribution to the advancement of knowledge on the use of machine learning in marketing.

Despite the fact that India and China are not recognized as benchmarks in terms of scholarly impact due to a relatively low number of citations, they stand out as key benchmarks in terms of scholarly productivity in the use of machine learning in marketing due to their high number of publications. A prominent example of India’s contribution is a study in the journal Marketing Intelligence & Planning, in which the authors explore the evolution of research in the field of relationship marketing from a machine learning perspective ( Das et al., 2022). This publication contributes to the existing body of knowledge and strengthens India’s scientific productivity in the field of machine learning applied to marketing.

In the case of China, we found a work in the journal Scientific Programming, in said study, the authors develop a luxury marketing model based on machine learning classification algorithms ( Chen et al., 2021), although the study has not received a high number of citations, demonstrates China’s contribution in the field of machine learning applied to marketing strategies and reinforces its scientific productivity in this area.

### Analysis of the thematic evolution of the use of machine learning in marketing

In the analysis of the thematic and conceptual evolution in the scientific literature on the use of machine learning in marketing, outstanding concepts were identified in different years, as shown in Figure 6. In 2006, the most studied approach was “Evolutionary Programming”, as a relevant example of contribution in that year is the work where the authors presented an intelligent approach to heart disease diagnosis using Machine Learning and Springleaf’s marketing response ( Dangi, Choudhury and Kumar, 2016).

Subsequently, in 2020, the most relevant concept was “big data”, which received considerable attention in the scientific literature. An outstanding study proposed an effective determination of marketing strategies based on customer segmentation using machine learning techniques ( Purnomo, Azzam & Khasanah, 2020). In addition, an approach based on machine learning to improve marketing in social networks was presented ( Arasu, Seelan & Thamaraiselvan, 2020).

In the years 2021, 2022 and 2023, the leading concept was “digital marketing”, which has been widely explored in the recent scientific literature. Notable examples include the evaluation of machine learning algorithms for marketing data analysis to predict grocery store sales ( Gopagoni, Lakshmi, & Chaudhary, 2020). Research on the application of artificial intelligence and neural networks in advertising and media has also been presented ( Bara et al., 2022). It also compared content marketing strategies of digital brands using machine learning ( Chen, 2023).

### Analysis of thematic clusters on the use of machine learning in marketing

Figure 7 shows the most prominent cluster, marked in red, which includes keywords such as “classification”, “automatic assistance”, “security”, “algorithm”, “data processing”, “data collection”, and “digital transformation”. This cluster highlights the importance of applying data classification and processing techniques to improve the security and effectiveness of marketing strategies in the digital environment ( Jayashree et al., 2022).

The second most relevant cluster, shown in green, includes keywords such as “data mining”, “data analytics”, “predictive analytics”, “naive Bayes”, “direct marketing”, “telecommunications”, “smote”, “online advertising", and "real-time bidding". This cluster highlights the importance of using data mining techniques and predictive analytics to improve the efficiency and accuracy of marketing strategies, especially in the area of online advertising and real time bidding ( Alhejaily et al., 2022; Bayoude et al., 2018).

The third most significant cluster, shown in purple, includes keywords such as “deep learning,” “social media marketing,” “tweets,” “sentiment analysis,” “display advertising,” and “neural networks. This cluster highlights the importance of deep learning and sentiment analysis in social media marketing, where neural network techniques are used to understand and exploit user-generated information on social media platforms ( Sharma et al., 2022; Tahmaz et al., 2020).

The fourth most important cluster, shown in light blue, includes keywords such as “digital marketing”, “prediction”, and “interpretation”. This cluster highlights the importance of using machine learning to predict and understand consumer behavior in the digital marketing environment, as well as to interpret the data and extract relevant knowledge for strategic decision making ( Sharma et al., 2021; Kaponis & Maragoudakis, 2022).

### Analysis of frequency and conceptual validity around the use of machine learning in marketing

**Figure 8.**
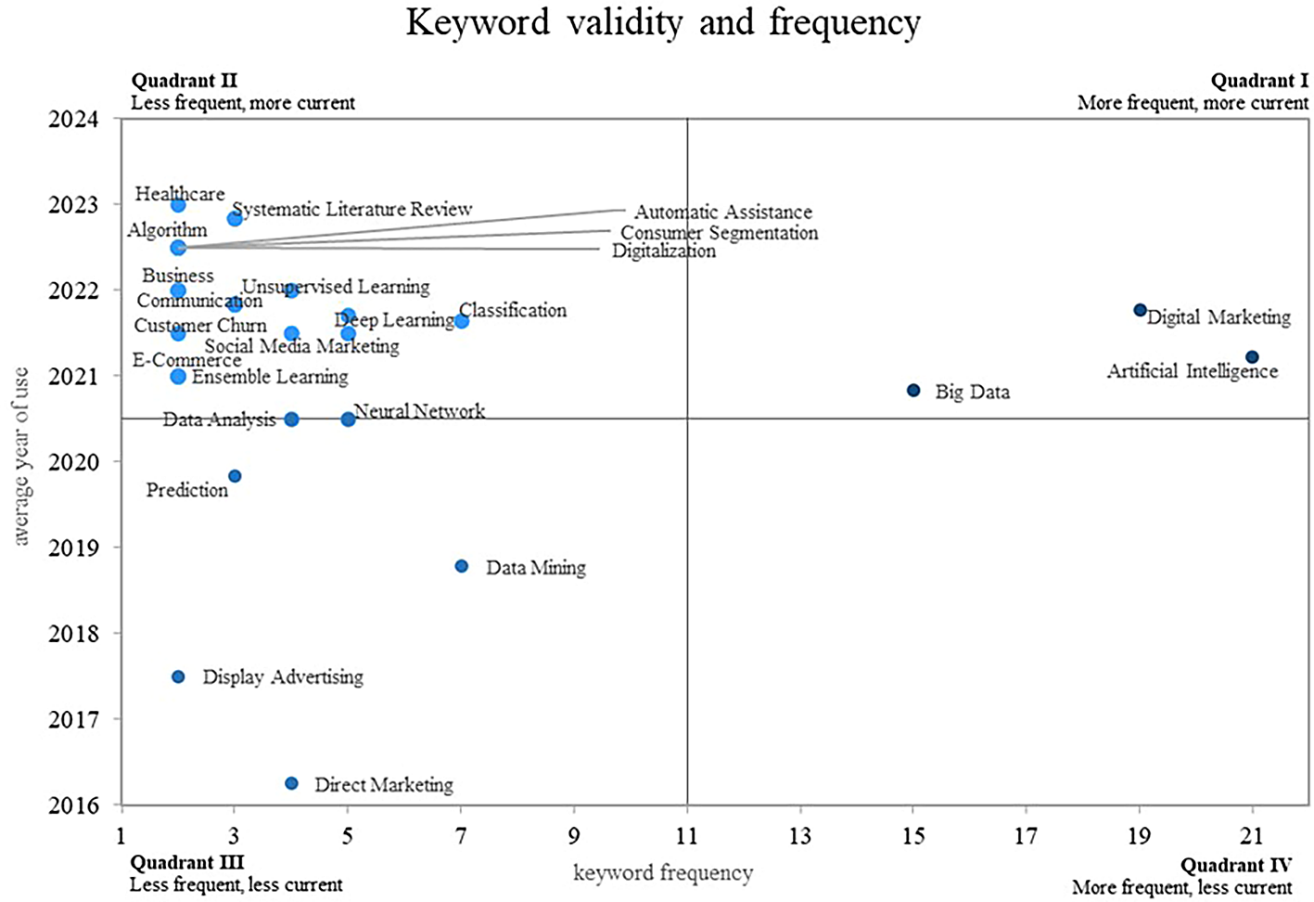
Keyword validity and frequency.

On the other hand, in quadrant 3 of Figure 8, there is a group of keywords that have a particular position. These keywords, which include Direct Marketing, Display Advertising, Data Mining and Prediction, do not appear as the most frequent terms, nor are they positioned as the most current in the scientific literature studied; this pattern suggests that although these topics are relevant, they have not yet reached full consolidation in the context of machine learning research in marketing.

There is also quadrant 2, through which the concepts are positioned that, although they do not account for a very high frequency of use, this has occurred in recent years, which is why they are considered emerging concepts within the field. research on the uses of machine learning in marketing. One of the prominent concepts in this quadrant is that of “Healthcare”, which has been addressed in recent works, an investigation examines the impact of Machine Learning on digital marketing techniques and its application in the healthcare system ( Kapula et al., 2022), the study highlights the relevance of the implementation of Machine Learning tools in the field of health, which demonstrates its growing importance today.

Another concept present in Quadrant 2 is that of ”systematic literature review”. One study provides a systematic review of the literature in the context of precision marketing, analyzing the application of machine learning ( Koufi, Belangour & Sadiq, 2023). This review highlights the need for rigorous and systematic research to better understand the role of machine learning in marketing and its impact on strategic decision making.

The term “automated assistance” is also included in this quadrant, and its importance lies in automating tasks and improving the customer experience. Research has examined the impact of Machine Learning and Big Data in the digital transformation of marketing, highlighting the role of Machine Learning in providing automated and personalized assistance to consumers ( Jain et al., 2022).

Another relevant concept in quadrant 2 is “consumer segmentation”, which has been studied in the field of marketing, the following article proposes methods based on machine learning and linear programming for efficient planning and selection of advertising in the media, highlighting the importance of consumer segmentation in marketing strategies ( Amgalanbaatar and Batnasan, 2022).

Finally, the concept of “digitalization” has also emerged as a relevant topic in Quadrant 2. In a published article, machine learning is used to analyze the effectiveness of the marketing mix in the context of social marketing, focusing on the activities of fashion brands on Twitter during the pandemic ( Xia and Liu, 2022). This study highlights the importance of digitalization in marketing strategies and the need to apply machine learning techniques to better understand its impact.

Finally, there is quadrant 1 of the Cartesian plane, through which there are concepts that are both the most common and the most current, which is why they are categorized as growing, leading and consolidated concepts within the scientific literature, one of these concepts is “Digital Marketing”, which has gained significant relevance today; The following researches highlight the application of machine learning in the expansion and efficiency of digital marketing campaigns, showing how the use of machine learning techniques allows the creation of similar audiences, the automatic tagging of content, and the improvement of the efficiency of content marketing ( Duvvuri, 2021) and ( Salminen et al., 2019), the growing adoption of digital marketing supports its importance today and its potential to drive the success of marketing strategies.

Another prominent concept in Quadrant 1 is “Artificial Intelligence”. Some publications highlight the importance of market segmentation through machine learning models based on Artificial Intelligence, especially in the area of customer relationship management and customer profitability accounting ( Florez-Lopez and Ramon-Jeronimo, 2009). This approach shows how the application of Artificial Intelligence in marketing can improve the accuracy and efficiency of market segmentation, resulting in more effective and customer-focused strategies.

In addition, the concept of “big data” is another key element in Quadrant 1. There are studies that address the adoption of analytical tools based on machine learning in the field of digital marketing ( Miklosik et al., 2019). Big data has become an invaluable source of information for marketing strategies, enabling the collection and analysis of large amounts of data to gain deep insights into consumer behavior and make evidence-based decisions.

### Classification of keywords on the use of machine learning in marketing according to their function

After analyzing the behavior of the keywords in this bibliometrics in terms of thematic evolution, clustering and classification of these in terms of their validity and frequency, Table 1 is presented, which contains the classification of the main keywords related to the use of Machine Learning in marketing based on their function.

**Table 1.** Classification of keywords by function.

Keyword	Related tools	Applications	Features
Healthcare	Predictive modeling	Personalized medicine	Improved patient outcomes
Systematic Literature Review	Text mining	Research synthesis	Efficient evidence evaluation
Algorithm	Decision trees	Customer segmentation	Automated pattern recognition
Automatic Assistance	Chatbots	Customer support	24/7 availability
Consumer Segmentation	Clustering analysis	Targeted marketing	Precise audience targeting
Digital Marketing	Recommendation engines	Customer engagement	Enhanced personalized campaigns
Artificial Intelligence	Natural language processing (NLP)	Sentiment analysis	Automated data interpretation
Big Data	Data mining	Market trend analysis	Scalable data processing

In accordance with the above, there are the keywords considered emerging in the field of research, in which healthcare, systematic literature review, algorithm, automatic assistance, consumer segmentation, digital marketing, artificial intelligence and big data are distinguished. Their classification serves as a basis for a key analysis, so that in the future other studies can base their contributions on them.

### Practical implications

This study, applied to the applications of machine learning in marketing, has revealed important practical implications. Conceptually, a thematic evolution has been observed in the research, moving from the initial focus on Evolutionary Programming to a greater analysis of aspects related to Neural Networks, Big Data and Digital Marketing. This transition indicates a paradigm shift in the way the study of these applications is approached, reflecting the growing importance of cutting-edge technologies in the field of marketing.

The analysis of thematic clusters has made it possible to identify the conceptual affinity between key terms such as “digital marketing”, “interpretation”, “prediction” and “healthcare”, suggesting that there is a significant interrelationship between digital marketing, data interpretation, behavior prediction and the application of machine learning in the field of healthcare; these findings have relevant practical implications for marketers, as they demonstrate the need to understand and use these emerging technologies to improve decision-making and the effectiveness of marketing strategies.

Keyword frequency and currency analysis revealed the emergence of new terms such as “healthcare”, “systematic literature review”, “algorithm”, “automated assistance” and “consumer segmentation”. These terms reflect the growing importance of the application of machine learning in healthcare, the need for systematic literature review, the development of specific algorithms, automatic assistance in marketing tasks, and consumer segmentation, and the findings suggest the need to update the knowledge and skills of marketers to adapt to these new concepts and take full advantage of the opportunities they offer.

### Limitations

This bibliometric study on machine learning applications in marketing, carried out according to the PRISMA-2020 methodology and based on the Scopus and Web of Science databases, has certain limitations that must be taken into account. First of all, the selection of the databases could have affected the completeness of the results, since there are other sources of scientific information that could be important for this study and that were not included in it.

Another important limitation lies in the use of specific tools such as Microsoft Excel ^®^ and VOSviewer ^®^ for bibliometric analysis; although these tools are widely used in the scientific community, their choice can influence the results and the interpretation of the bibliometric indicators obtained. In addition, it is necessary to take into account that the analysis of keywords and their co-occurrence may underestimate the complexity of the topics covered in the articles, since it does not take into account the complete framework in which the words are used.

Finally, it is important to recognize that bibliometrics is based on existing scientific publications and is therefore subject to the limitations inherent in previous studies. These limitations may include the lack of representation of unpublished or ongoing research, as well as possible biases in the selection of articles analyzed. In addition, bibliometrics cannot provide an exhaustive assessment of the scientific quality of articles because it is based on bibliometric indicators that may be influenced by other indicators.

### Research gaps

After analyzing the results and the corresponding discussion, Table 2 is proposed, which shows a series of research gaps or conceptual gaps recognized in the research on the use of machine learning in marketing, its justification and questions for future research that could be useful in mitigating existing gaps.

**Table 2.** Research gaps.

Category	Gaps	Justification	Questions for future researchers
Thematic gaps	1. Integrating AI and ML into SME marketing strategies.	The research has mainly focused on large enterprises and general applications, leaving aside the context of SMEs.	How can machine learning techniques be adapted and applied to specific marketing strategies for SMEs?
	2. Personalize the customer experience in marketing.	More focus is needed on personalizing the customer experience in the digital environment using machine learning, as this can significantly improve marketing strategies.	What are the most effective ways to personalize the customer experience in digital marketing using machine learning?
Geographic gaps	1. Applications of ML in marketing in developing countries.	Most research has focused on developed countries, leaving a gap in knowledge about machine learning applications in marketing in developing countries.	What are the specific barriers and opportunities for implementing marketing machine learning applications in developing countries?
	2. Comparative analysis of the application of ML in marketing.	There is a need for comparative analysis across geographic regions to understand differences in the adoption and use of machine learning in marketing.	What are the differences in the adoption and effectiveness of machine learning applications in marketing across geographic regions?
Interdisciplinary gaps	1. Integration of psychological theories into ML models.	There is a gap in the integration of psychological theories in the development of machine learning models used in marketing.	How can psychological theories be integrated into the development of machine learning models to improve marketing strategies?
	2. Collaboration between marketing experts and machine learning.	There is a lack of collaboration between marketing experts and machine learning experts, which limits the use of the potential of these technologies.	What are the best practices for fostering collaboration between marketers and machine learning experts on joint research projects?
Temporary gaps	1. Long-term evaluation of the impact of ML applications in marketing.	A deeper understanding of the lasting impact of machine learning applications in marketing is needed through long-term evaluations.	What is the long-term impact of implementing machine learning applications on marketing strategies?
	2. Research on the future of machine learning in marketing.	Forward-looking research is needed to analyze the trends and future of machine learning in marketing.	What are the emerging technologies and future trends in machine learning that will impact marketing?

### Research agenda

**Figure 9.**
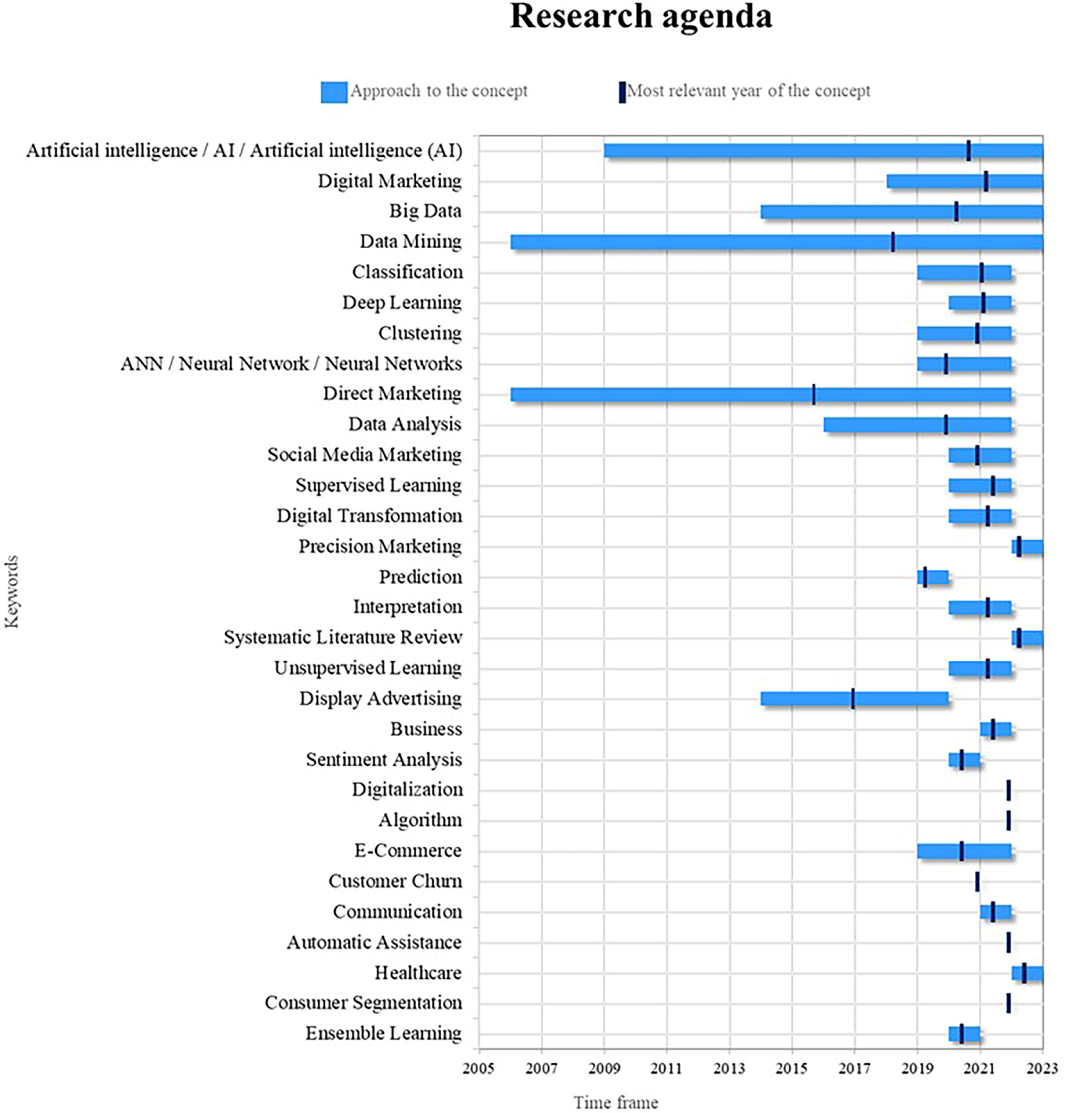
Research agenda.

Finally, in Figure 9, a proposed research agenda for this bibliometrics is presented with the idea that other researchers can use it as a guide for future scientific studies based on topics considered as trends, emerging and current. For this purpose, two important points are examined: (1) the time in which the term has been addressed in the literature and (2) the year of greatest relevance in academic production, the latter indicating the period in which the concept has had a greater role in academic production and at the same time that it has been studied in the last year.

Based on the results and discussion of this bibliometric analysis, a research agenda is proposed that aims to guide new research in the field of the uses of Machine Learning in marketing. In this sense, in relation to Artificial Intelligence, its current importance in the uses of Machine Learning in marketing lies in its ability to improve the personalization of marketing strategies and decision making based on data. In future research, the development of more advanced machine learning algorithms that take full advantage of the capabilities of artificial intelligence can be further explored, as well as natural language processing techniques to improve the understanding of consumer preferences and needs. In addition, it is crucial to investigate how artificial intelligence can be used to analyze large amounts of data and extract useful knowledge to support strategic marketing decisions.

Regarding data mining, its current importance in the use of machine learning in marketing focuses on the ability to discover hidden patterns and relationships in massive data sets, in future research, it can be investigated in the development of algorithms of more sophisticated data mining that allow the identification of specific market segments and the personalization of marketing strategies in real time, likewise, sequence and association mining techniques can be explored to discover patterns of consumer behavior and emerging trends in the market. market, it is essential to investigate how to make the most of the available data to obtain valuable information and improve the effectiveness of marketing strategies.

In the case of direct marketing, the current importance of machine learning in marketing lies in its ability to create direct and personalized connections with customers. In future research, it is possible to deepen the development of automatic learning algorithms that allow for more precise segmentation and more effective communication in direct marketing, you can investigate how to use machine learning to identify consumer behavior patterns and offer personalized offers and messages based on your individual preferences, it is also relevant to explore the integration of direct marketing with other digital marketing techniques and process automation to improve the efficiency and impact of direct marketing campaigns.

Digital marketing has become an emerging and highly relevant term in the field of using machine learning in marketing; currently, digital marketing has experienced exponential growth thanks to the advancement of digital technologies and the availability of massive data. In future studies, the use of Machine Learning can be deepened to improve personalization and segmentation in marketing strategies. digital marketing, as well as the application of machine learning algorithms to optimize the effectiveness of online advertising campaigns.

Big Data is another emerging term that plays a fundamental role in the context of machine learning applied to marketing; the massive availability of data and processing capacity have opened up new opportunities for extracting valuable information and making decisions based on data. New research can explore how machine learning can help analyze and extract meaningful insights from large data sets, as well as develop predictive and recommendation models to better understand consumer behavior and personalize marketing strategies.

Precision marketing is an emerging term that refers to the ability of companies to reach specific audiences with personalized and relevant messages; in the context of machine learning in marketing, one can explore how machine learning algorithms can be used to identify patterns and individual characteristics of consumers in order to offer personalized experiences adapted to their needs and preferences; in addition, the application of machine learning techniques can be explored in market segmentation and identification of specific niches with the aim of optimizing marketing strategies and maximizing return on investment.

The systematic review of literature is a key research methodology to analyze and synthesize existing knowledge on a specific topic; in the context of Machine Learning in Marketing, a systematic review of literature can be conducted to identify trends, advances, and challenges. In the application of machine learning in different areas of marketing, this review can serve as a basis to identify gaps in the literature and establish new lines of research, as well as provide an overview of best practices and recommendations in the use of machine learning in marketing.

Deep learning is a machine learning technique that has gained a lot of attention in recent years due to its ability to process and understand complex data such as images, text, and speech. In the area of machine learning applications in marketing, one can explore how deep learning can improve the understanding of consumer behavior from unstructured data, such as social network interactions, customer comments, and online reviews. In addition, the application of deep learning in the automatic generation of advertising content and in the detection of patterns and emerging trends in the market can be explored.

Clustering is a machine learning technique that is used to group similar data into categories or segments, in the field of applications of machine learning in marketing, clustering plays a crucial role by allowing the identification of customer groups with characteristics and similar behaviors, future research could explore how to apply clustering in market segmentation and personalization of marketing strategies, identifying specific groups of consumers with similar needs and preferences, also the combination of clustering techniques with other machine learning techniques, such as recommendation and prediction, can be explored to develop more accurate recommendation systems and more effective marketing strategies.

Social media marketing has experienced exponential growth in recent years, becoming a fundamental part of the marketing strategies of companies, in reference to the use of machine learning, the analysis of social networks and the extraction of valuable information from them. have become essential, in future studies, you can explore how to use machine learning techniques to analyze large volumes of data generated in social networks, identify patterns of consumer behavior and track emerging trends, likewise, you can investigate how to harness the power of machine learning to optimize the management of marketing campaigns on social networks and improve user interaction and participation.

Healthcare is an emerging field in which Machine Learning has great potential to drive the personalization of marketing strategies in the field of health, in future research, it can be explored how to use Machine Learning algorithms to analyze large data sets. clinical data and generate valuable insights about patient behavior, treatment preferences and the effectiveness of medical interventions, in addition, you can explore how to take advantage of Machine Learning to develop personalized recommendation systems in the field of health, offering patients receive relevant information tailored to their individual needs, this research can help improve the quality of healthcare services and promote more accurate and efficient medical care.

Prediction is a fundamental aspect of the use of machine learning in marketing. It allows companies to anticipate the needs and preferences of consumers in order to optimize their strategies and decisions. Currently, prediction has become even more relevant with the increased availability of data and the development of more sophisticated algorithms. However, new studies could increase its importance by exploring more advanced approaches, such as the use of deep learning techniques and graph-based predictive models; these advances could improve the accuracy of predictions, allowing companies to adapt more effectively to market changes and offer personalized experiences to consumers.

Sentiment analysis is a key technique in machine learning applied to marketing, as it allows us to understand consumers’ opinions, attitudes, and emotions toward a brand, product, or service. Currently, sentiment analysis has become an invaluable tool for businesses, providing them with valuable insight into their customers’ perceptions and enabling them to take proactive steps to improve their satisfaction, however new studies could reinvigorate its relevance by investigating more advanced sentiment analysis approaches such as Using models based on recurrent neural networks and natural language processing, such approaches could improve the accuracy and understanding of consumer sentiment, allowing businesses to tailor their marketing strategies more effectively and strengthen the emotional connection with their audiences.

Ensemble learning, or joint learning, is a powerful technique in machine learning that combines multiple learning models to produce more accurate and robust predictions. In the context of marketing, ensemble learning can be particularly beneficial by providing insight into more complete and reliable information about consumer behavior and preferences; likewise, new studies could revitalize its importance by exploring innovative approaches in creating and combining learning models together; for example, future research could investigate the implementation of ensemble learning algorithms based on deep learning techniques or the use of genetic algorithms to optimize the combination of models; these advances could drive the widespread adoption of ensemble learning in marketing, improving the accuracy of predictions and allowing companies to make more informed and strategic decisions.

## Conclusion

Firstly, a growing interest in machine learning applications in marketing is observed in 2019, 2020, 2021 and 2022, indicating an upward trend in academic and professional attention to this particular field, reflecting its relevance today.

Secondly, there is an exponential growth in the number of scientific articles related to machine learning applications in marketing, this increase demonstrates a constant and ever-growing interest in the subject, suggesting that the research and adoption of this technology continues to evolve rapidly.

Third, several key referents in the field were identified, the authors Miklosik and Evans, along with IEEE Access and Journal of Business Research, stand out as prominent figures in the research on machine learning applications in marketing, in addition, a significant international scientific collaboration is observed, indicating an exchange of knowledge and collaboration in the academic community. Therefore, it is concluded that these referents are key to establish a solid theoretical base and to further promote research on this topic.

Finally, several thematic clusters and relevant scientific associativity were identified; the thematic evolution shows a change from an initial focus on evolutionary programming to more contemporary topics such as big data and digital marketing; likewise, the “digital marketing” thematic clusters, “interpretation”, “prediction”, and “healthcare” stand out as areas of greatest conceptual affinity and relevance in current research.

Overall, these conclusions indicate that machine learning applications in marketing are a constantly growing and evolving field, with significant interest in the scientific community and an increasingly focused focus on emerging and relevant topics. These findings provide a solid foundation for future research and suggest that the study of this topic will continue to be of great importance in the future.

## Ethics and consent

Ethical approval and Consent were not required.

## Data availability statement

Underlying data: No data are associated with this article.

### Extended data

Zenodo: Dataset and supporting materials for the study “Applications of Machine Learning (ML) in the context of marketing: a bibliometric approach”. https://doi.org/10.5281/zenodo.8436315 ( Cardona-Acevedo et al., 2023).

The project contains the following underlying data:

• Scopus _ ML in marketing.csv
• Web of Sciences _ ML in marketing.csv (Raw data supporting the findings of this study).

The data and materials are publicly available under a Creative Commons Attribution 4.0 International license.

### Reporting guideline

Zenodo: Dataset and supporting materials for the study “Applications of Machine Learning (ML) in the context of marketing: a bibliometric approach”. https://doi.org/10.5281/zenodo.8436315 ( Cardona-Acevedo et al., 2023).

PRISMA Checklist.pdf (Checklist detailing compliance with PRISMA 2020 guidelines).

The data and materials are publicly available under a Creative Commons Attribution 4.0 International license.
