# Supplementary material for: Applications of Machine Learning (ML) in the context of marketing: a bibliometric approach
Source: F1000Res. 2025 Mar 17;14:92. Originally published 2025 Jan 15. [Version 2] doi: 10.12688/f1000research.160010.2 (PMC11926528; doi:10.12688/f1000research.160010.2)
Supplement: Peer review history

# Reviewer report: Reviewer response for version 2

**DOI:** 10.5256/f1000research.179211.r371292

**Published:** 2025-03-20

**Authors:** Ravi Shankar Rai

**Affiliations:** 1 India and Education and Training Division, Noida, India

The authors have addressed the concerns raised and made revisions in the article. The article is may be proceed for the indexing.

Are the rationale for, and objectives of, the Systematic Review clearly stated?

Yes

Is the statistical analysis and its interpretation appropriate?

Yes

If this is a Living Systematic Review, is the ‘living’ method appropriate and is the search schedule clearly defined and justified? (‘Living Systematic Review’ or a variation of this term should be included in the title.)

Not applicable

Are sufficient details of the methods and analysis provided to allow replication by others?

Yes

Are the conclusions drawn adequately supported by the results presented in the review?

Partly

Reviewer Expertise:

Data analysis, Entrepreneurship development, Marketing analytics

I confirm that I have read this submission and believe that I have an appropriate level of expertise to confirm that it is of an acceptable scientific standard.

# Reviewer report: Reviewer response for version 1

**DOI:** 10.5256/f1000research.175812.r366958

**Published:** 2025-03-10

**Authors:** Suryo Hadi Wira Prabowo

**Affiliations:** 1 Universitas Negeri Malang, Malang, East Java, Indonesia

The rationale, objectives, and systematic review have been clearly presented in the article. The methods and analysis are well-explained in detail, making them easy to study and replicate for future researchers. The application of statistical analysis and interpretation is appropriate for dissemination. The conclusions align with the topic addressed by the author. Since this is not a Living Systematic Review, there is no need to elaborate on the scheduling definition. Based on these considerations, I accept this article for further processing.

Are the rationale for, and objectives of, the Systematic Review clearly stated?

Yes

Is the statistical analysis and its interpretation appropriate?

Yes

If this is a Living Systematic Review, is the ‘living’ method appropriate and is the search schedule clearly defined and justified? (‘Living Systematic Review’ or a variation of this term should be included in the title.)

Not applicable

Are sufficient details of the methods and analysis provided to allow replication by others?

Yes

Are the conclusions drawn adequately supported by the results presented in the review?

Yes

Reviewer Expertise:

Marketing, Digital Marketing, AI in Marketing, Targeted Marketing

I confirm that I have read this submission and believe that I have an appropriate level of expertise to confirm that it is of an acceptable scientific standard.

# Reviewer report: Reviewer response for version 1

**DOI:** 10.5256/f1000research.175812.r361017

**Published:** 2025-02-26

**Authors:** Ravi Shankar Rai

**Affiliations:** 1 India and Education and Training Division, Noida, India

The paper presents a detailed bibliometric analysis of machine learning (ML) applications in marketing, emphasizing the rising interest and exponential rise in research papers between 2006 and 2023. The analysis highlights major trends, notable authors, journals, nations, topic clusters, and new keywords. The article provides a comprehensive overview of the application of **machine learning (ML)** in **marketing**, highlighting its potential to transform marketing strategies through data analysis, consumer behavior prediction, and campaign optimization.

**Strengths of paper:**

 a. The combination of quantitative (e.g., publication counts, citations) and qualitative metrics e.g. keyword analysis offers a comprehensive picture of the research environment.

 b. The study clearly depicts the progression of research issues, from evolutionary programming to modern problems like as big data, digital marketing, and neural networks.

 c. The study clearly depicts the development of research issues, from evolutionary programming to current problems like as big data, digital marketing, and neural networks.

 d. The discussion of practical applications of machine learning in marketing, such as consumer behavior prediction, campaign optimization, and personalized marketing, helps to bridge the gap between academics and industry.

**Area for improvements:**

 a. Issues like data privacy, algorithmic bias, or the societal impact of ML in marketing may be added in ethical implications.

 However, the article only briefly touches on **ethical and privacy concerns**, which are critical in the context of ML-driven marketing. Expanding on these concerns would significantly strengthen the article by addressing the following aspects:

**1. Ethical Use of Predictive Analytics**

- **Why It Strengthens the Article**: Predictive analytics, a key application of ML in marketing, raises ethical concerns about **consumer manipulation** and **privacy invasion**.
- **How to Incorporate**: The article could discuss the ethical boundaries of predictive analytics. For example, it could explore the difference between **personalization** and **manipulation**, emphasizing the need for marketers to use predictive analytics responsibly and transparently.

**2. Impact on Vulnerable Populations**

- **Why It Strengthens the Article**: ML-driven marketing can disproportionately affect vulnerable populations, such as children, the elderly, or low-income individuals, who may be more susceptible to targeted advertising or manipulation.
- **How to Incorporate**: The article could discuss the ethical responsibility of marketers to avoid exploiting vulnerable groups. For instance, it could explore the use of **age verification systems** to prevent targeted advertising to children or the implementation of **ethical guidelines** to ensure marketing practices do not take advantage of vulnerable consumers.

**3. Data Privacy and Security**

- **Why It Strengthens the Article**: The use of ML in marketing relies heavily on consumer data, raising significant privacy concerns. Unauthorized access or misuse of this data can lead to breaches of privacy and security.
- **How to Incorporate**: The article could explore the importance of **data anonymization** and **encryption** to protect consumer information. It could also discuss the ethical responsibility of marketers to ensure that data is stored securely and used only for its intended purpose. For instance, the article could cite examples of companies that have faced backlash for data breaches and how they could have implemented better security measures.

As per my understanding, by incorporating a detailed discussion of **ethical and privacy concerns**, the article would provide a more balanced and comprehensive view of the application of ML in marketing. Addressing these concerns would not only strengthen the article's relevance but also highlight the importance of responsible and ethical practices in the rapidly evolving field of ML-driven marketing. This would make the article more valuable to both researchers and practitioners, encouraging them to consider the ethical implications of their work and adopt best practices to protect consumer rights and trust.

Are the rationale for, and objectives of, the Systematic Review clearly stated?

Yes

Is the statistical analysis and its interpretation appropriate?

Yes

If this is a Living Systematic Review, is the ‘living’ method appropriate and is the search schedule clearly defined and justified? (‘Living Systematic Review’ or a variation of this term should be included in the title.)

Not applicable

Are sufficient details of the methods and analysis provided to allow replication by others?

Yes

Are the conclusions drawn adequately supported by the results presented in the review?

Partly

Reviewer Expertise:

Data analysis, Entrepreneurship development, Marketing analytics

I confirm that I have read this submission and believe that I have an appropriate level of expertise to confirm that it is of an acceptable scientific standard, however I have significant reservations, as outlined above.

# Response

**Published:** 2025-03-10

**Authors:** JHOANY ALEJANDRO VALENCIA ARIAS

We thank the reviewers for their kind and valuable suggestions. The updated version, which contains the following changes, is attached:

**Comment**: a. Issues like data privacy, algorithmic bias, or the societal impact of ML in marketing may be added in ethical implications.

**Response**: Ethical implications section added in the discussion, addressing data privacy, algorithmic bias, and societal impact of ML in marketing.

**Comment:** "1. Ethical Use of Predictive Analytics Why It Strengthens the Article: Predictive analytics, a key application of ML in marketing, raises ethical concerns about consumer manipulation and privacy invasion. How to Incorporate: The article could discuss the ethical boundaries of predictive analytics. For example, it could explore the difference between personalization and manipulation, emphasizing the need for marketers to use predictive analytics responsibly and transparently."

**Response**: Section added discussing ethical boundaries in predictive analytics, distinguishing between personalization and manipulation while ensuring responsible marketing practices.

**Comment:** "2. Impact on Vulnerable Populations Why It Strengthens the Article: ML-driven marketing can disproportionately affect vulnerable populations, such as children, the elderly, or low-income individuals, who may be more susceptible to targeted advertising or manipulation. How to Incorporate: The article could discuss the ethical responsibility of marketers to avoid exploiting vulnerable groups. For instance, it could explore the use of age verification systems to prevent targeted advertising to children or the implementation of ethical guidelines to ensure marketing practices do not take advantage of vulnerable consumers."

**Response: **Section added on protecting vulnerable groups from ML-driven marketing manipulation through ethical guidelines and age verification measures.

**Comment: "**3. Data Privacy and Security Why It Strengthens the Article: The use of ML in marketing relies heavily on consumer data, raising significant privacy concerns. Unauthorized access or misuse of this data can lead to breaches of privacy and security. How to Incorporate: The article could explore the importance of data anonymization and encryption to protect consumer information. It could also discuss the ethical responsibility of marketers to ensure that data is stored securely and used only for its intended purpose. For instance, the article could cite examples of companies that have faced backlash for data breaches and how they could have implemented better security measures."

**Response**: Section added covering data privacy, encryption, anonymization, and ethical responsibilities in securing consumer information to prevent breaches.
